# Network-based approach identifies key genes associated with tumor heterogeneity in HPV positive and negative head and neck cancer patients

**DOI:** 10.1038/s41598-025-13604-0

**Published:** 2025-08-07

**Authors:** Sumeet Patiyal, Piyush Agrawal

**Affiliations:** 1 Cancer Data Science Laboratory, National Cancer Institute, NIH, Bethesda, MD 20814 USA; 2 Division of Medical Research, Research Centre, SRM Medical College Hospital, SRM Institute of Science and Technology, Kattankulathur, Chennai India

**Keywords:** Network-based approach, Head and neck cancer, Human papilloma virus, Tumor heterogeneity, Differentially expressed genes, Cancer hallmarks, Cancer, Computational biology and bioinformatics

## Abstract

Head and Neck Squamous Cell Carcinoma (HNSCC) is the seventh most prevalent cancer worldwide and is classified as human papillomavirus (HPV) positive or negative. Substantial heterogeneity has been observed in the two groups, posing a significant clinical challenge. In the disease context, global transcriptional changes are likely driven by a few key genes that reflect the disease etiology more accurately compared to differentially expressed genes (DEGs). We implemented our network-based tool PathExt on 501 TCGA-HNSCC samples (64 HPV positive & 437 HPV negative) to characterize central genes in two subtypes, where in subtype 1, HPV-positive samples were considered as cases and negative as controls, and vice versa in subtype 2. We also identified DEGs and performed several analyses on multiple benchmarking datasets to compare the biology of central genes with DEGs. PathExt key genes performed better with respect to DEGs in both subtypes in recapitulating disease etiology. Gene ontology analysis using central genes revealed shared biological processes such as “epithelial cell proliferation” as well as subtype-specific processes (immune- and metabolic-related processes in subtype 1 and peptide-related processes in subtype 2). However, in the case of DEGs, no subtype-specific processes were seen. Additionally, PathExt central genes did better than DEGs on external validation datasets that were specific to HNSCC and included HNSCC-specific cancer driver genes, FDA-approved therapeutic targets, and pan-cancer tumor suppressor genes. Unlike DEGs, central genes exhibit significant expression in various cell types, enrichment for cancer hallmarks, and mutated protein systems. Central gene expression-based machine learning model shows better performance than DEGs in classifying responders/non-responders with 0.74 AUROC. Lastly, the top 10 potential therapeutic targets and drugs were proposed. Overall, we observed PathExt as a complementary approach to DEGs, characterizing common and HNSCC subtype-specific key genes associated with distinct HNSCC molecular subtypes.

**Supplementary Information:**

The online version contains supplementary material available at 10.1038/s41598-025-13604-0.

## Introduction

Head and Neck Squamous Cell Carcinoma (HNSCC) is the seventh most prevalent cancer worldwide^1^. HNSCC is a group of malignancies that results from the head and neck squamous cell linings. As per GLOBOCAN estimates, HNSCC accounts for ~ 4.5% of the global death toll, with 890,000 new cases and 450,000 deaths per annum^2^. The incidence rate of HNSCC varies as per geographical region, with India leading the chart and accounting for around 80% of all the cases. One of the main causes of these rising cases is due to chewing of the areca nut, which is carcinogenic in nature^3^.

Human papillomavirus (HPV) infection has been seen in the HNSCC, based on the detection of HPV DNA, notably HPV-16 it can be broadly categorized into two subtypes: HPV-positive (HPV-Pos) and HPV-negative (HPV-Neg). These two categories differ significantly in their molecular profile, etiopathogenesis, and clinical outcomes. Among the two subtypes, the HPV-Neg subtype accounts alone for ~ 75% of the cases and has a significantly worse prognosis compared to the HPV-Pos subtype. Treatment options include surgery, chemotherapy, radiotherapy, and targeted agents such as EGFR monoclonal antibody (mAb) inhibitors and two PD-1 inhibitors; however, the overall response rates have been moderate^4,5^. Hence, there is a need to identify novel drug targets for better health outcomes.

Tumor heterogeneity has been a serious concern and a major problem limiting the success rate of targeted therapies and survival outcomes in patients^6^. Similar observations have been made in the case of HNSCC as well, where integration of the HPV genome into the host genome is associated with HPV + HNSCC initiation and progression^7^. Authors reported biased distribution of integration breakpoints across the HPV genome, four HPV states, among which 49% of the tumor progression was without HPV genome integration. Likewise, Qin et al. show the heterogeneity in gene expression, copy number mutation, driving mutations, etc., and how the HPV-positive and HPV-negative subtypes are different^8^.

Previous studies largely relied on differentially expressed genes (DEGs) to characterize new therapeutic targets; however, recent studies have shown that DEGs-based approaches have major limitations. First, individual genes are subject to stochastic variability in gene expression, which limits their reproducibility and consistency. Secondly, DEGs do not capture the key mediators of the global transcriptomic changes, such as transcription factors (TFs), kinases, and other regulatory proteins, and largely focus on the downstream targets. Therefore, there is a pressing need to identify these potential mediators causing global transcriptomic changes. Recently, we and others have shown that network-based approaches could be an alternative to traditional differential expression-based approaches^9–12^. In our recent papers, we have implemented our network-based tool PathExt, which identifies differentially active pathways using a knowledge-based network. From these differentially active pathways, it further identifies corresponding central genes in the subnetwork (also known as TopNet). These central genes are more robust than DEGs and provide a more mechanistic view of potential mediators of transcriptional changes across conditions as shown in our recent studies^10,12^.

We implemented the PathExt^13^ framework on the TCGA-HNSCC datasets (HPV-Pos and HPV-Neg) to characterize PathExt central genes mediating global transcriptomic changes in the two subtypes. We also characterized the DEGs in both the subtypes and compared the performance with PathExt. We did several analyses and found that PathExt identified central genes that were consistent across HNSCC subtypes. This improved our previous understanding of how tumors are different from each other.

## Methodology

### Data generation and PathExt central genes identification

Transcriptomic data for 501 primary head and neck squamous cell carcinoma (HNSCC) patients of The Cancer Genome Atlas (TCGA) were downloaded from the UCSC Xena Browser^14^. Among these 501 samples, 64 samples were HPV positive and remaining 437 samples were HPV negative. We created two subtypes using these samples, where in Subtype 1, HPV-positive samples were taken as case and HPV-negative samples as control, and in Subtype 2, HPV-negative samples were taken as case and HPV-positive samples as control. For both subtypes, we characterized the differentially expressed genes (DEGs) using the ‘limma’ R package^15^ and selected the top 100 central genes with the highest log-fold change and adjusted *p*-value (FDR) of < = 0.05. Next, we used our network-based tool PathExt^13^ to characterize key upregulated genes in one subtype compared to other. For each gene in the sample, we calculated the node weight. Our previous papers^12,13^ provide a detailed description of the PathExt framework, but here we provide a brief sketch.

In the original PathExt paper^13^ simple log-fold change in expression of a gene in a case sample compared to a control has been taken as an input. However, the major limitation with such approach is that it can overemphasize genes with high variability or low expression, leading to noisy or non-robust network results. It does not account for baseline gene variability, making it prone to false positives Given the dependence of the magnitude of log(fold change) on the expression level, as done in our recent studies, we computed the expected log(fold change) for given control gene expression. Next, we took the difference between the observed and expected log fold change and considered it as node weight. We used ‘Loess fit’ using R package to find the expected absolute log (fold change) by regressing absolute log (fold change) values for all genes against gene expression in control samples. This new method has been validated in our recent previous studies^10,12^.

The matrix of the node weights for each gene in each sample is provided as an input to the PathExt, which maps these genes to an experimentally defined protein-protein interaction (PPI) network and characterizes the most differentially active paths. We perform this iteration 200 times to ensure that statistical significance remains intact. Finally, we provide an output in the form of TopNet, which represents the significant differential paths. We further computed ripple centrality scores from this TopNet file and use the genes with the highest scores for further analyses. In our case, we selected the top 100 genes based on centrality score in each sample and further computed the frequency of the genes. Lastly, we selected the top 100 genes with the highest frequency for further analyses. We termed these genes as ‘central genes’ throughout the study. This approach was followed in our recent papers^12^where detailed descriptions have been provided.

In this study, we performed the analysis in two manners. In the first approach, we considered subtype 1 i.e., HPV positive samples as ‘case’ and HPV negative samples as ‘control’ and in the second approach, we considered subtype 2 i.e., HPV negative samples as ‘case’ and HPV positive samples as ‘control’.

### Gene ontology enrichment analyses

To characterize enriched biological processes, we used the ClusterProfiler 4.0 package^16^ where the top 100 DEGs or PathExt central genes as foreground, and the background was the default gene lists used by the software. The database used by the software was human database with minimum and maximum gene size was set 10 and 500 respectively, to ensure specific terms. We used the function ‘simplify’ and “Wang” as a measure with a cutoff value of 0.8 and FDR ≤ 0.05 as a statistical measure to remove the redundant terms.

### Comparison of PathExt central genes and DEGs with benchmark genesets and other network-based method MOMA

We compiled several previously published genesets to assess the overlap of identified PathExt central genes and DEGs. We curated the list of cancer driver genes specific to HNSCC from previously published databases: (a) IntoGen^17^ and (b) DriverDBv3^18^. Next, we curated the list of tumor suppressor genes, and the list of targets targeted by the FDA-approved drugs for HNSCC from the CancerDrugs_DB database^19^. We also curated the list of 1665 human specific transcription factors (TFs) from AnimalTFDB3.0^20^ and 521 kinases from KinHub databases^21^. We used Fisher’s exact test as a measure of the statistical test and reported Odds Ratio (OR), where OR > 1 represents enrichment and FDR ≤ 0.05 represents significance. Lastly, we performed the comparative analysis of PathExt with another network-based approach called MOMA (Multi-Omics Master-Regulator Analysis). The tool identifies cancer specific and in general 407 master regulator (MR) proteins which regulates the key cancer hallmarks and predict survival outcomes in patients^22^.

### Enrichment analysis of various cancer hallmark and functionally mutated protein systems

We downloaded the cancer hallmarks from the MSigDB^23^ in the form of ‘.gmt’ file and computed the enrichment of our PathExt central genes and DEGs in them. For the enrichment analysis, we used the “enricher” function in the ClusterProfiler 4.0 package. We displayed the significant terms (defined as – log10 (adj.*p*-value) ≥ 1.3) in the form of a heatmap using the ComplexHeatmap package^24^ in R. Likewise, we downloaded the 395 protein systems (each system comprises a list of certain genes) that have been significantly mutated across cancer types from the NeST database^25^. Next, we overlap our gene sets with the NeST systems, and the ones with significant overlap (FDR ≤ 0.05) were reported.

### Cell type specific gene expression analysis using single cell data

We downloaded the publicly available single-cell transcriptome data for HNSCC patients from the Gene Expression Omnibus with the ID GSE181919^26^. We only used the primary cancer samples for our analyses, as there were samples from metastatic, normal, and leukoplakia classes. We implemented the Seurat pipeline^27^ and the standard procedure for processing the data and obtaining the gene expression in each cell and cell type. Firstly, we computed the mean expression and variance across cell types and performed one-way ANOVA^28^ across cell types for both DEG and PathExt gene sets. We further normalized the data using z-scoring and computed the log (observed/expected) fraction of genes expressed in each cell type, as done in our previous paper^12^.

### Machine learning analysis to classify responder and non-responder

We used the scikit-learn Python package^29^ for developing machine learning (ML) models that can classify responders and non-responders to a given treatment. In total, we developed five different ML models, namely support vector machine (SVM), random forest (RF), adaboost, gradient boost, and extratree (ET). The TCGA-HNSCC dataset was used to build the models. Gene expression was used as a feature, and different techniques’ parameters were tweaked to find the best ones using GridSearch technique. The feature selection process was conducted prior to and independent of any use of outcome labels during model training and evaluation. Five-fold cross-validation technique was performed to ensure the robustness of the model, where the whole data is divided into five parts. Out of these five parts, four parts are used for model training, and one is used for testing. The process is repeated five times until all the set is used for testing, and lastly, average results are reported. We routinely use this standard approach^30,31^.

Post internal validation, we further validated the performance of the above-trained models on an independent dataset by Bossi et al.,^32^ where HNSCC patients were treated by cetuximab. While training, for subtype 1, we considered HNSCC-HPV positive as the positive class and HPV-negative as the negative class and vice versa for subtype 2. We computed performance using the Area Under Receiver Operating Characteristics (AUROC) measures and generated a plot using the pROC R package^33^ Area Under the Precision-Recall Curve (AUPRC) and F1-score. To ensure statistical robustness and accuracy of the models, we implemented bootstrap sampling technique^34^ (*n* = 1000 times) and computed the performance. DeLongs test was also implemented to check the statistical significance of the models^35^.

### Identifying key therapeutic targets and drug repurposing analysis

To obtain the potential drug targets in each subtype, we first created the protein-protein interaction (PPI) network using the STRING database^36^ with confidence score > 0.7. Next, we imported the created network into the Cytoscape^37^ and use the ‘cytoHubba plugin’^38^ to get the hub genes. Top 10 hub genes were selected based on the ‘Degree’ node score calculation method implemented in the software which is a standard approach as done in previous studies^39,40^. Next, we map the top 10 targets with the CMap database^41^maintained by BROAD Institute to obtain the potential drugs. CMap database provides the list of drugs which are FDA approved or under clinical trials against a given target or gene. We also used the database DGIdb 5.0^42^ to investigate the drug-gene interaction between our potential genes and the mapped drugs against them. List of the genes were provided as an input to the server and the results were downloaded in the form of ‘.tsv’ file.

## Results

### Summary of the study

**Fig. 1:**
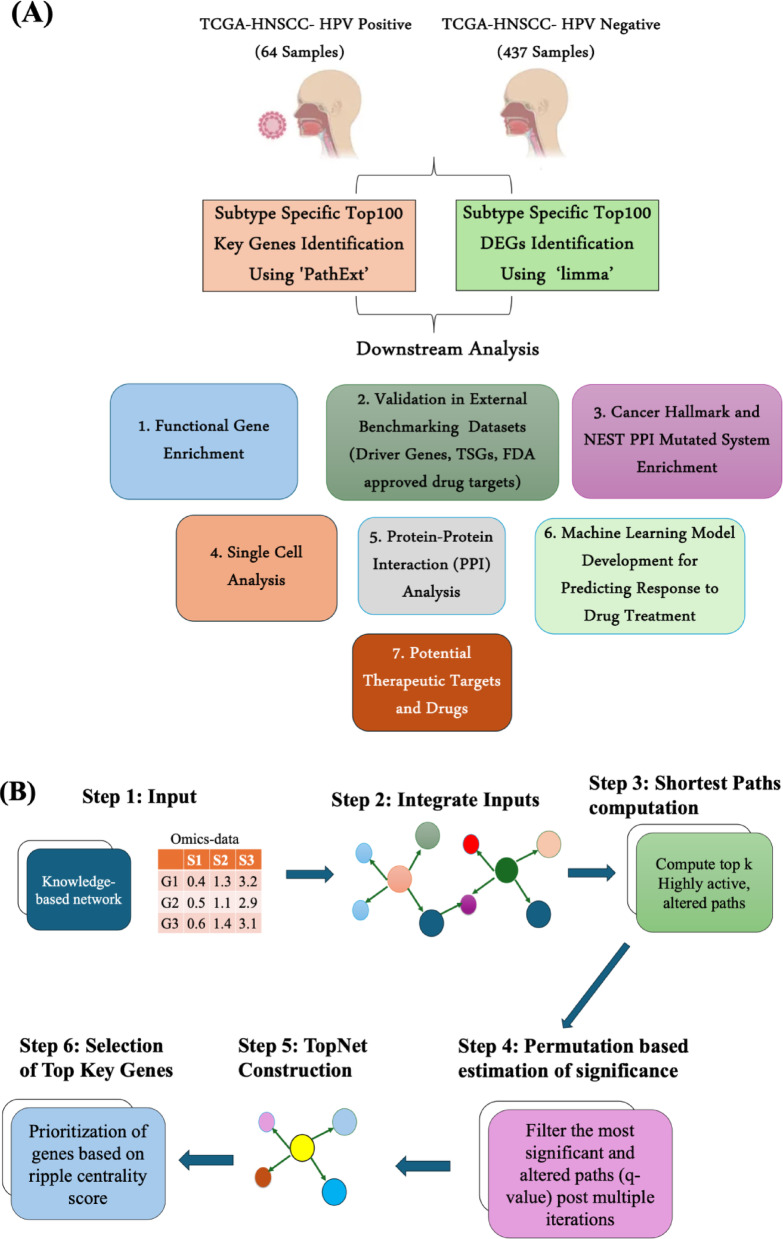
Workflow of the current study. (**A**) The figure represents the overall approach implemented in this study to perform comparative study between PathExt and DEGs based approaches. (**B**) A flowchart of PathExt pipeline used to characterize key genes from each sample is provided.

In this study, we conducted a comparison between our network-based approach, PathExt, and the state-of-the-art method of using DEGs to understand cancer biology. We characterized the top 100 activated PathExt central genes using PathExt as well as the top 100 upregulated DEGs in subtype 1 (HPV-positive HNSCC patients) compared to subtype 2 (HPV-negative HNSCC patients). Similarly, we characterized the top 100 upregulated PathExt central genes and top 100 upregulated DEGs in subtype 2 compared to subtype 1. We performed various analyses using these 4 sets of genes (top100 PathExt activated PathExt central genes and top100 upregulated DEGs in each subtype), including enriched biological processes, enriched cancer hallmarks, mutated protein systems in cancer, benchmarking with several external validation datasets, cell types expressing the genes using single-cell analyses, ML models to classify responder and non-responders to treatment, novel therapeutic targets, and potential drugs against them. We showed that PathExt central genes were far better than DEGs in capturing the heterogeneity between HNSCC HPV-Positive and HPV-Negative samples and recapitulates the potential biological mechanism driving the cancer. Figure 1A illustrates the overall architecture of the present study and Fig. 1B illustrates the PathExt pipeline.

### PathExt reflects putative HNSCC subtype specific biology compared to DEGs

The following analyses are based on the top 100 PathExt activated PathExt central genes and upregulated DEGs characterized in HNSCC subtype 1 and subtype 2.

Inter-sample transcriptional heterogeneity is a global challenge, making treatment difficult or ineffective for the majority of the population. DEGs usually provide the information about the downstream genes affected post-event and do not capture the genes associated with global transcriptome changes. However, PathExt is more context-specific and focuses more on upstream regulators (including transcription factors) rather than the affected downstream genes and provides much better therapeutic options. The tool promises to characterize genes that are consistent across samples.

In the case of subtype 1, PathExt captures significantly altered networks in 58/64 samples. Out of 58 samples, *TP53* was present in 21 samples (~ 36%), followed by *UBC* in 20 samples (34%); *CTNNB1* and *ACTB* in 19 samples (33%). In the case of DEGs, *ARHGAP4* was the top gene. When we looked at the potential role of the above genes, we observed that PathExt-identified genes, for example, *TP53*, recapitulate the cancer biology in HNSCC HPV-positive patients. More than 70% of HNSCC patients have reported mutations in *TP53*^43^ that are associated with a good prognosis for HNSCC with HPV-positive classification^44^. However, DEGs have not demonstrated a direct role for *ARHGAP4* in HNSCC patients, particularly in HPV-positive samples. Similarly, in subtype 2, PathExt significantly captured 305 samples. Among these 305 samples, *TGFB1* appeared in 153 samples (50%), followed by *EGFR* in 148 samples (48.52%) at 2nd place and *MAPK1* in 143 samples (46.88%), whereas in the case of DEGs, *SPRR2G* shows the highest LogFC value, followed by *KRTDAP* and *KLK5*. Bedi et al. in their study show that the elevated role of *TGFB1* within the tumor microenvironment (TME) is associated with evading response to therapy and developing resistance^45^. However, when we looked for the *SPRR2G* role in HNSCC HPV-negative patients, we did not find enough information supporting its role in HNSCC, specifically in subtype 2. The frequency of all the genes characterized by PathExt for subtypes 1 and 2 is provided in Supplementary Tables S1 and S2 whereas the differential expression results by limma is provided in Supplementary Table S3 and S4 for subtype 1 and 2 respectively.

**Fig. 2:**
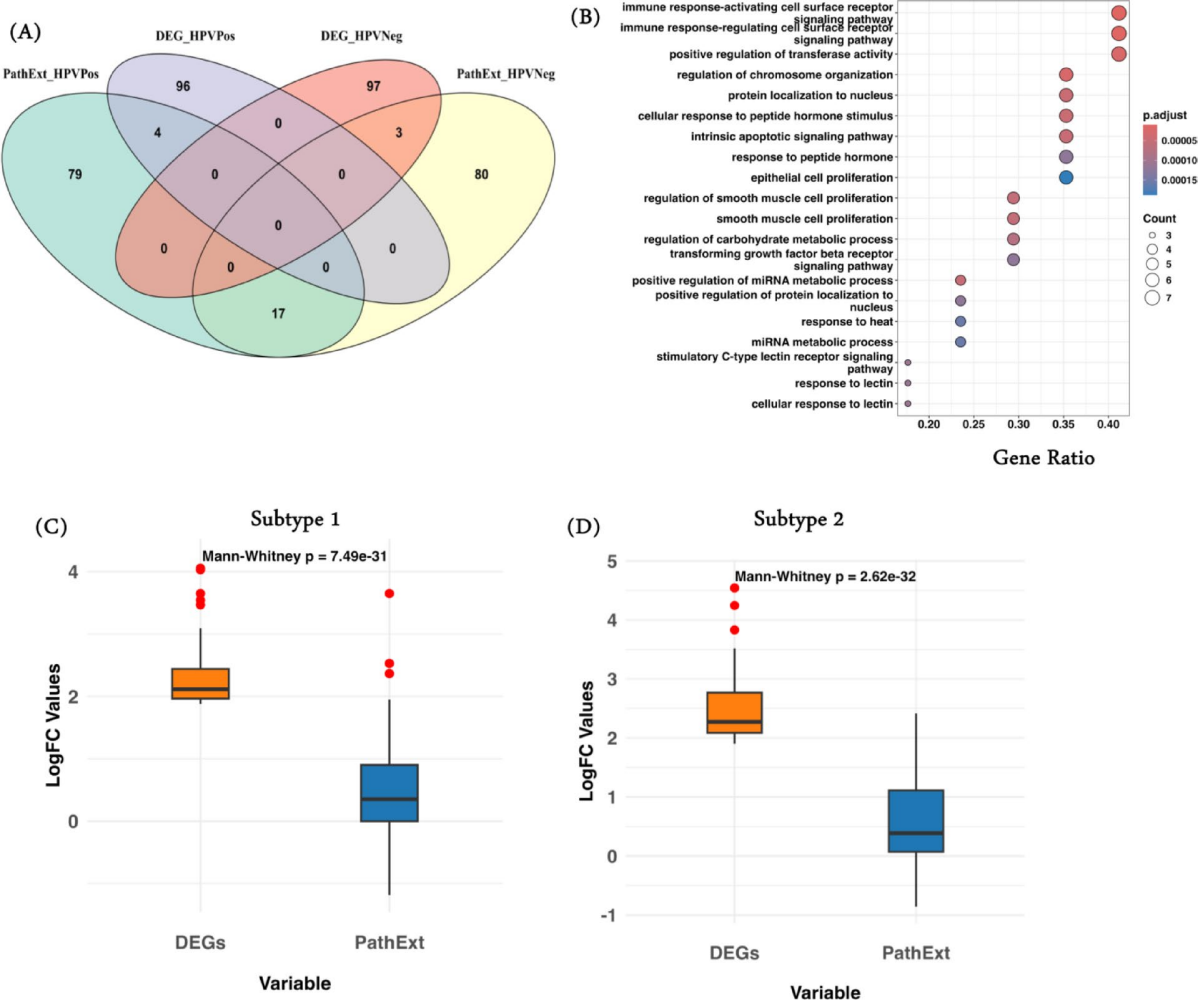
PathExt capture biologically relevant genes. (**A**) Venn diagram of top100 PathExt central genes and DEGs characterized in subtype 1 and subtype 2 showing common and unique genes; (**B**) Top20 enriched significant biological processes associated with common 17 PathExt central genes among subtype 1 and 2; (**C**) Log fold change comparison of gene expression (case compared to control) for the top100 PathExt central genes and DEGs characterized in subtype 1; (**D**) Log fold change comparison of gene expression (case compared to control) for the top100 PathExt central genes and DEGs characterized in subtype 2.

Our gene overlapping analysis revealed an interesting observation: we found 17 common genes (Supplementary Table S5) among the top 100 PathExt central genes of both subtype 1 and subtype 2, and zero among DEGs (Fig. 2A). When we looked at the enriched processes associated with the common genes, we observed enrichment of processes such as “immune response-activating cell surface receptors,” “epithelial cell proliferation,” etc., which are in general in nature and can be present in both subtypes (Fig. 2B). Complete list of enriched processes is provided in Supplementary Table S6. Previous findings also supported this observation^46,47^; however, in the case of DEGs, we do not find any similar genes. We also identified a few common genes between PathExt central genes and DEGs, demonstrating PathExt’s significant ability to capture genes with both high and low log fold change values (Fig. 2C, D).

**Fig. 3:**
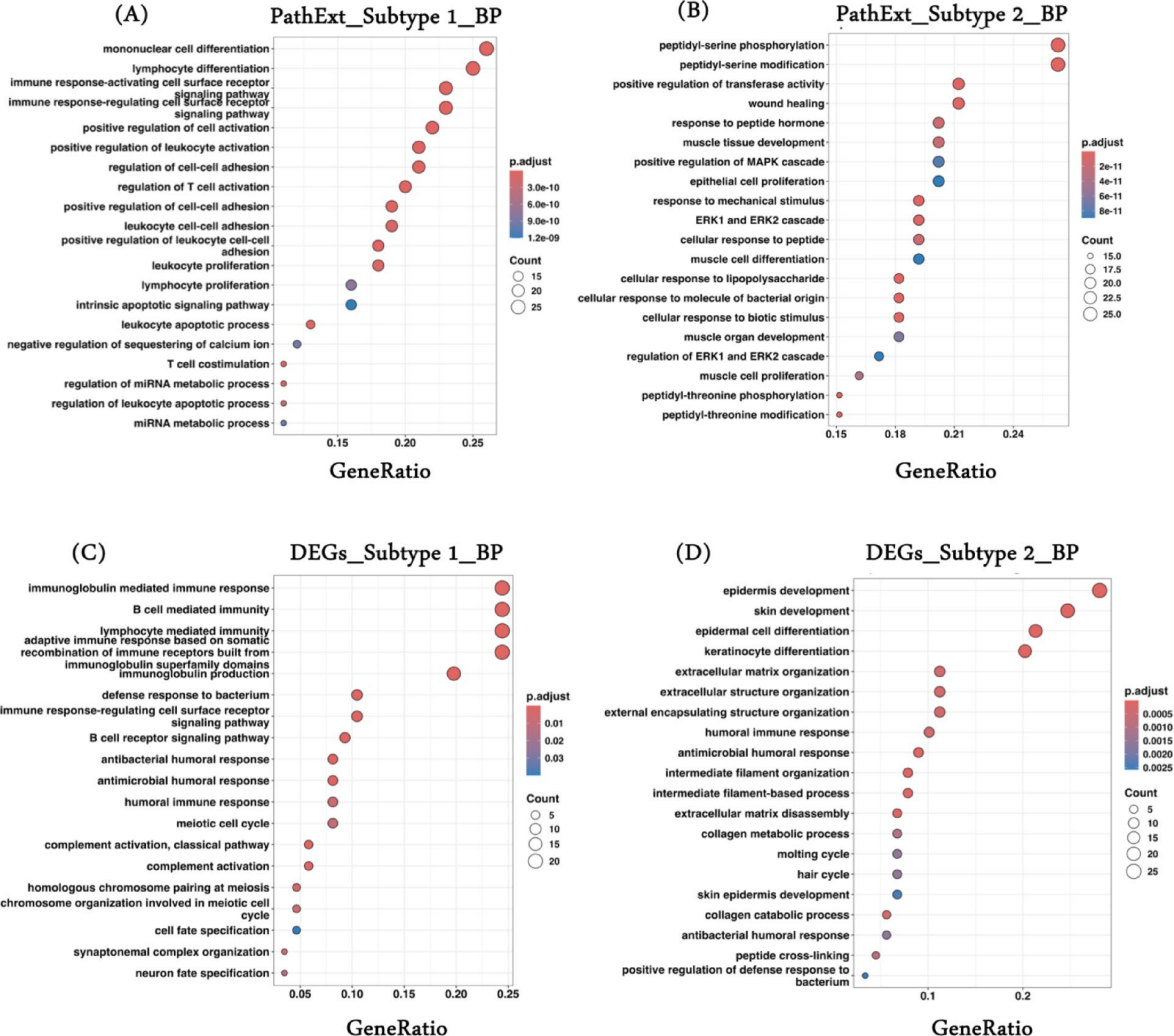
PathExt central genes shows distinct processes in HNSCC subtypes. ClusterProfiler 4.0 package was used to obtain significant enriched terms associated with the top100 PathExt key genes and DEGs. Here, we have shown top20 enriched biological processes associated with (**A**) top100 PathExt central genes in subtype 1; (**B**) top100 PathExt central genes in subtype 2; (**C**) top100 upregulated DEGs in subtype 1; (**D**) top100 upregulated DEGs in subtype 2. Here “Gene Ratio” is the fraction of the input genes in the GO term and ‘Count’ represents the number of key genes common in the gene list of the given term. Only significant processes (FDR ≤0.05) are shown here.

In addition, we looked for the enriched biological processes associated with the top 100 PathExt central genes and DEGs characterized in subtypes 1 and 2. PathExt central genes found in subtype 1 were mostly involved in immune-related processes like “immune response-activating cell surface receptor,” “regulation of T-cell activation,” and metabolic processes like “regulation of miRNA metabolic processes” and “mRNA metabolic processes” (Fig. 3A). On the other hand, PathExt central genes found in subtype 2 were linked to peptide-related processes like “peptidyl-serine phosphorylation,” “peptidyl-serine modification,” and “response to peptide hormone” as well as cell-signaling processes like “ERK1 and ERK2 cascade” and “cellular response to lipopolysaccharide” (Fig. 3B). Our findings were specifically enriched for HNSCC HPV negative patients, and we found sufficient independent studies supporting our above observation^48–50^.

We performed similar analyses with the top 100 DEGs in subtype 1 and subtype 2 and observed enrichment of mostly similar processes; for example, in subtype 1 and subtype 2, DEGs were enriched majorly for processes such as “humoral immune response” and “defense response to bacterium.”. DEGs also have some distinct processes, such as in subtype 2, such as “skin development,” “hair cycle,” etc. (Fig. 3C, D). Overall, PathExt central genes show distinct processes in subtypes compared to DEGs, reflecting heterogeneity in the processes separating the subtypes. A complete list of all enriched processes associated with the top 100 PathExt central genes and DEGs for subtypes 1 and 2 is provided in Supplementary Tables S7–S10.

### PathExt outperformed DEGs in capturing cancer associated hallmarks, mutated PPI systems, driver genes, tumor suppressor genes, and therapeutic targets

PathExt’s main strength is to capture genes that are not differentially expressed but are associated with regulating global transcriptome changes. These genes reflect the true picture of the disease biology; hence they become better therapeutic targets. Several separate studies have described these genes, pointing out their role in cancer as driver genes, tumor suppressors, and therapeutic targets against several FDA-approved drugs. Later, researchers compiled such information in the form of databases for public use.

**Fig. 4:**
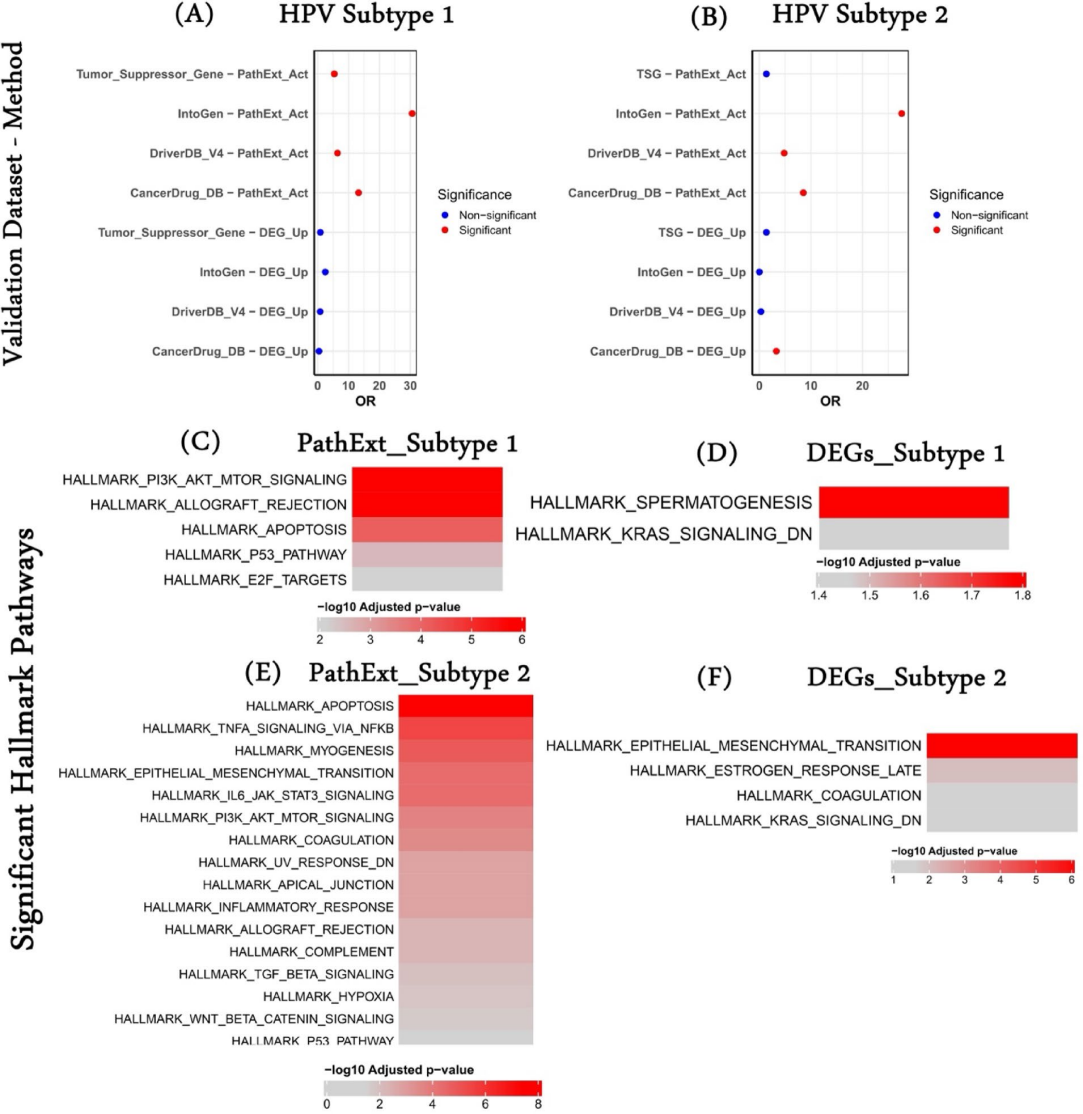
Comparative performance of PathExt and DEGs on cancer hallmarks and external datasets. (**A**) Fisher’s odds ratio showing the ability of subtype 1 top100 PathExt central genes and upregulated DEGs in recapitulating HNSCC specific PathExt central genes in multiple external datasets. (**B**) Fisher’s odds ratio showing the ability of subtype 2 top100 PathExt central genes and upregulated DEGs in recapitulating HNSCC specific PathExt central genes in multiple external datasets. (**C**) Enriched cancer hallmarks associated with subtype 1 top100 PathExt central genes. (**D**) Enriched cancer hallmarks associated with subtype 1 top100 upregulated DEGs. (**E**) Enriched cancer hallmarks associated with subtype 2 top100 PathExt central genes. (**F**) Enriched cancer hallmarks associated with subtype 2 top100 upregulated DEGs.

Here, we assess the overlap ability of our gene sets (PathExt and DEGs) to see if they successfully recapitulate such information. First, we overlapped our gene sets with driver genes specific to HNSCC from two databases, IntoGen and DriverDB v3, and observed that PathExt significantly outperformed DEGs in both subtypes and achieved an odds ratio (OR) of up to 30. Next, we overlap the gene sets with the FDA-approved drug targets and observe similar performances. Finally, we compared PathExt’s PathExt central genes and DEGs with the TSGs (pan-cancer because HNSCC-specific is not available) and saw that PathExt did better than DEGs in subtype 1. However, in subtype 2, neither method showed a significant overlap (Fig. 4A–B).

Next, we compared our method with another network-based method called MOMA (See Methods). We obtained the list of 140 master regulators (MRs) specific to head and neck cancer and perform overlap analysis with the above-mentioned external datasets. As shown in Supplementary Figure S1, PathExt key genes from both subtypes outperformed MOMA in recapitulating genes from the external benchmarking datasets. PathExt genes shows high Odd’s ratio compared to MOMA, computed using Fisher’s exact test.

Next, we wanted to see the enrichment of various cancer hallmarks associated with PathExt central genes and upregulated DEGs in a subtype-specific manner. We downloaded the hallmark data from MSigDb and performed the enrichment analysis. As shown in Fig. 4C, subtype 1 PathExt central genes were enriched for several hallmarks such as “PI3K-AKT_MTOR_Signaling,” “Apoptosis,” “P53_targets,” etc. However, subtype 1 top 100 DEGs were enriched for only 2 hallmarks, “Spermatogenesis” and “KRAS_Signaling_DN” (Fig. 4D). We found that subtype 2-specific PathExt central genes exhibited a higher number of hallmarks than subtype 1. They were majorly enriched for the signaling hallmarks highlighting the role of signaling processes in HNSCC patients with HPV-Negative classification. We also observed some other key hallmarks, such as “inflammatory response,” “apoptosis,” and “hypoxia” (Fig. 4E). In the case of DEGs, we observed enrichment of fewer hallmarks only (Fig. 4F).

In addition to cancer hallmarks, we also analyzed the enrichment of significantly mutated PPI systems in multiple cancer types curated by the Ideker group in the form of a database, commonly known as NeST. We obtained data for 395 systems, where each system comprises a list of significantly mutated genes. We overlap our PathExt central genes and DEGs of both subtypes with each system gene list. We performed enrichment analysis and extracted the list of systems that were significantly overlapping (FDR of ≤ 0.05). In the case of subtype 1, the PathExt central genes were significantly enriched for multiple systems, as shown in Table 1, whereas DEGs didn’t show any significant enrichment. In the case of subtype 2, PathExt genes show significant overlap with multiple systems (Table 2), whereas DEGs show enrichment of only fewer systems (Table 3).

**Table 1:** Subtype 1 PathExt key gens significant enrichment in nest PPI systems.

Terms	*p*-value	Overlap	Term size	Top genes count	Adj. *p*-value
Cyclin D associated events in G1	5.12E-12	10	77	100	2.02E-09
CDK holoenzyme complex II	1.33E-08	6	33	100	5.25E-06
Regulation of CDK activity	1.56E-15	13	96	100	6.16E-13
Cell cycle arrest	1.87E-19	14	67	100	7.36E-17
CDK holoenzyme complex I	1.17E-10	7	30	100	4.61E-08
G1 to S cell cycle control	1.03E-10	6	16	100	4.07E-08
Regulation of E2F transcription via DREAM complex	1.68E-09	6	24	100	6.62E-07
Oncogene induced senescence	6.98E-10	5	10	100	2.75E-07
MDM2-p53 pathway	1.89E-07	4	11	100	7.45E-05
Transmembrane receptor tyrosine kinase signaling	5.05E-19	17	143	100	1.99E-16
DNA metabolic process	9.14E-06	8	206	100	3.60E-03
Erb and JAK-STAT signaling	5.17E-05	4	41	100	2.04E-02
TP53 regulates transcription of cell cycle genes	2.18E-09	5	12	100	8.57E-07
Extended beta-catenin destruction complex	2.58E-05	3	12	100	1.02E-02
MAPK and MAP2K activation	1.90E-05	4	32	100	0.00748585
PIK3-EGFR signaling	7.70E-07	4	15	100	0.00030332
Transcriptional regulation by TP53 via histone acetyltransferase	4.06E-08	4	8	100	1.60E-05
MAPK signaling	1.05E-08	7	55	100	4.15E-06
Regulation of TP53 Activity	2.82E-11	7	25	100	1.11E-08
Cytoplasm and extracellular space	8.18E-09	51	4880	100	3.22E-06
Nucleus and ribosome	7.34E-10	47	3957	100	2.89E-07
AKT/MTOR pathway II	9.37E-05	3	18	100	0.03692444
Immune system	2.46E-09	25	1276	100	9.68E-07
Regulation of biogenesis	2.54E-07	33	2630	100	0.00010021
Cell cycle	1.86E-18	26	554	100	7.31E-16
CBP and p300 binds NF-kB complex	3.52E-09	5	13	100	1.39E-06
Regulation of transcription	2.48E-06	22	1461	100	0.00097601
Ribonucleoprotein complexes II	8.26E-05	11	541	100	0.03254141
HDACs deacetylate histones	1.46E-05	4	30	100	0.00574887
Androgen receptor signaling pathway	1.25E-14	9	29	100	4.91E-12
PML body	6.95E-08	5	22	100	2.74E-05
Regulation of immune responses	1.39E-07	14	487	100	5.49E-05
Lymphocyte activation	1.14E-14	17	258	100	4.51E-12
H3 acetylation and H3K4 methylation crosstalk	5.28E-05	3	15	100	0.02081474
T cell receptor complex	3.34E-05	3	13	100	0.01317907
Other signaling processes	9.24E-11	37	2391	100	3.64E-08

List of mutated PPI systems which shows significant enrichment of subtype 1 PathExt central genes with system gene lists.

*Here Terms represents the mutated systems present in NeST database; ‘Overlap’ represents the number of overlap genes; ‘Term size’ is the number of genes present in the mutated system; ‘Top Genes Count’ is the number of PathExt central genes which is 100; and ‘Adj. *p*-value’ is the computed statistical value showing the enrichment.

**Table 2:** Subtype 2 PathExt key genes significant enrichment in NEST PPI systems.

Terms	*p*-value	Overlap	Term size	Top genes count	Adj. *p*-value
Transmembrane receptor tyrosine kinase signaling	4.03E-22	19	143	100	1.59E-19
ECM-cytoskeleton assembly	4.80E-14	12	95	100	1.89E-11
Regulation of CDK activity	5.47E-14	12	96	100	2.15E-11
Cytoplasm and extracellular space	1.64E-13	59	4880	100	6.48E-11
MAPK signaling	7.11E-12	9	55	100	2.80E-09
Extracellular matrix	2.88E-11	13	204	100	1.13E-08
Cell cycle	2.62E-10	18	554	100	1.03E-07
Actin filaments	4.93E-10	10	121	100	1.94E-07
Extracellular matrix organization	5.22E-10	15	373	100	2.06E-07
Cell motility	4.06E-09	30	1859	100	1.60E-06
MAPK and MAP2K activation	1.10E-08	6	32	100	4.31E-06
G1 to S cell cycle control	1.18E-08	5	16	100	4.65E-06
CDK holoenzyme complex II	1.33E-08	6	33	100	5.25E-06
Other signaling processes	2.58E-08	33	2391	100	1.02E-05
RAS-RAF-MAPK signaling	4.06E-08	4	8	100	1.60E-05
Contractile fiber	4.21E-08	11	246	100	1.66E-05
Cell cycle arrest	4.30E-08	7	67	100	1.69E-05
Erb and JAK-STAT signaling	5.24E-08	6	41	100	2.06E-05
Actin filament-based process	2.23E-07	11	290	100	8.77E-05
Cytoskeleton	2.38E-07	16	677	100	9.38E-05
Contractile fiber parts	2.55E-07	6	53	100	0.00010043
TP53 regulates transcription of cell cycle genes	2.82E-07	4	12	100	0.00011127
Collagen related ECM components	3.19E-07	6	55	100	0.0001258
CDK holoenzyme complex I	3.65E-07	5	30	100	1.44E-04
PIK3-EGFR signaling	7.70E-07	4	15	100	0.00030332
Actin and actin-associated proteins	1.36E-06	6	70	100	0.0005356
Structural constituent of muscle	1.42E-06	11	350	100	0.0005606
Cyclin D associated events in G1	2.39E-06	6	77	100	9.42E-04
Integrin binding	4.18E-06	3	7	100	0.00164843
Microfibril-collagen assembly	6.67E-06	3	8	100	0.0026279
Transcriptional regulation by TP53 via histone acetyltransferase	6.67E-06	3	8	100	0.0026279
Regulation of TP53 Activity	6.87E-06	4	25	100	0.00270506
PIK3CA-actomyosin complex	3.34E-05	3	13	100	0.01317907
AKT1 activation	3.34E-05	3	13	100	0.01317907
CBP and p300 binds NF-kB complex	3.34E-05	3	13	100	0.01317907
Extended PRC1 complex	8.90E-05	4	47	100	0.03505657
AKT/MTOR pathway II	9.37E-05	3	18	100	0.03692444
EGF/FGF stimulation of cell proliferation	1.11E-04	3	19	100	0.04368874

List of mutated PPI systems which shows significant enrichment of subtype 2 PathExt key genes with system gene lists.

*Here Terms represents the mutated systems present in NEST database; ‘Overlap’ represents the number of overlap genes; ‘Term size’ is the number of genes present in the mutated system; ‘Top Genes Count’ is the number of PathExt key genes which is 100; and ‘Adj. *p*-value’ is the computed statistical value showing the enrichment.

**Table 3:** Subtype 2 upregulated DEGs significant enrichment in NEST PPI systems.

Terms	*p*-value	Overlap	Term size	Top genes count	Adj. *p*-value
Keratinization	1.08E-45	38	267	100	4.25E-43
Extracellular matrix organization	2.63E-06	11	373	100	1.04E-03
Cornification	1.61E-44	30	114	100	6.35E-42
Other signaling processes	5.61E-15	43	2391	100	2.21E-12

List of mutated PPI systems which shows significant enrichment of subtype 2 PathExt key genes with system gene lists.

*Here Terms represents the mutated systems present in NeST database; ‘Overlap’ represents the number of overlap genes; ‘Term size’ is the number of genes present in the mutated system; ‘Top Genes Count’ is the number of PathExt central genes which is 100; and ‘Adj. p-value’ is the computed statistical value showing the enrichment.

### PathExt genes are enriched for transcription factors and kinases

One of the key points of PathExt based approach is its ability to capture upstream regulators (including transcription factors) rather than the affected downstream genes and provides much better therapeutic options. First, we looked at the enriched biological processes and GO terms associated with TFs and kinases. We observed enrichment of terms such as “Regulation of ncRNA transcription”, “Regulation of miRNA transcription”, “Epigenetic regulation of gene expression”, “Positive regulation of DNA binding”, “Regulation of transcription involved in G1/S transition”, “Histone acetylation” etc. in subtype 1, and “positive regulation of miRNA transcription”, “miRNA transcription”, “regulation of ncRNA transcription”, “transcription initiation-coupled chromatin remodeling”, etc. in subtype 2. Similarly, we looked for the terms enriched for kinase activity and we found terms such as “positive regulation of kinase activity”, “regulation of protein serine/threonine kinase activity”, etc. in subtype 1 and terms such as “positive regulation of kinase activity”, “transmembrane receptor protein serine/threonine kinase signaling pathway”, etc. in case of subtype 2. When we looked at the enriched terms for DEGs, we didn’t find any relevant terms associated to transcription and kinase.

We further downloaded the list of 1665 human TFs from AnimalTFDB 3.0 and 521 human kinases from the KinHub database and computed the proportion of top100 PathExt and DEGs in them. We performed Fisher’s exact test and computed odds ratio to show the significance. As shown in Table 4, PathExt showed significant proportions of TFs and kinases (except for TF in subtype 2) among the top100 key genes compared to DEGs, highlighting the efficiency of the PathExt to characterize TFs and kinases which are often missed by the DEGs.

**Table 4:** Enrichment of human transcription factors and kinases.

Method	Category	Count	Proportion	Odds Ratio	*p*-value
Subtype 1					
PathExt	TF	17	0.17	2.26842321	0.00354452
PathExt	Kinase	12	0.12	5.19494553	1.13E-05
DEGs	TF	7	0.07	0.82813858	0.73667074
DEGs	Kinase	1	0.01	0.37645688	0.92907791
Subtype 2					
PathExt	TF	10	0.10	1.22	0.32
PathExt	Kinase	36	0.36	22.52	1.19 × 10⁻³¹
DEGs	TF	3	0.03	0.34	0.99
DEGs	Kinase	0	0.00	0	1.00

Overlap Analayis was done to compute the proportion of the human TFs and kinases in the genesets (Top100 PathExt genes and DEGs) in both the subtypes. Fishers exact test was performed to compute the odds ratio and significance.

We also performed the overlap analyses of human TFs and kinases with the MOMA MRs and observed that it performed slightly better than PathExt in recapitulating TFs, however it failed when it comes to human kinases (Supplementary Figure S1).

Lastly, we created a TF-TF regulatory network using Trrust tool^51^. List of TFs was provided as an input, and the tool provided the network among the TFs. TF-TF regulatory network was obtained only in the case of PathExt and not DEGs and is provided as Supplementary Figures S2 and S3.

### Single cell analyses reveal high expression of PathExt central genes in tumor microenvironment

**Fig. 5:**
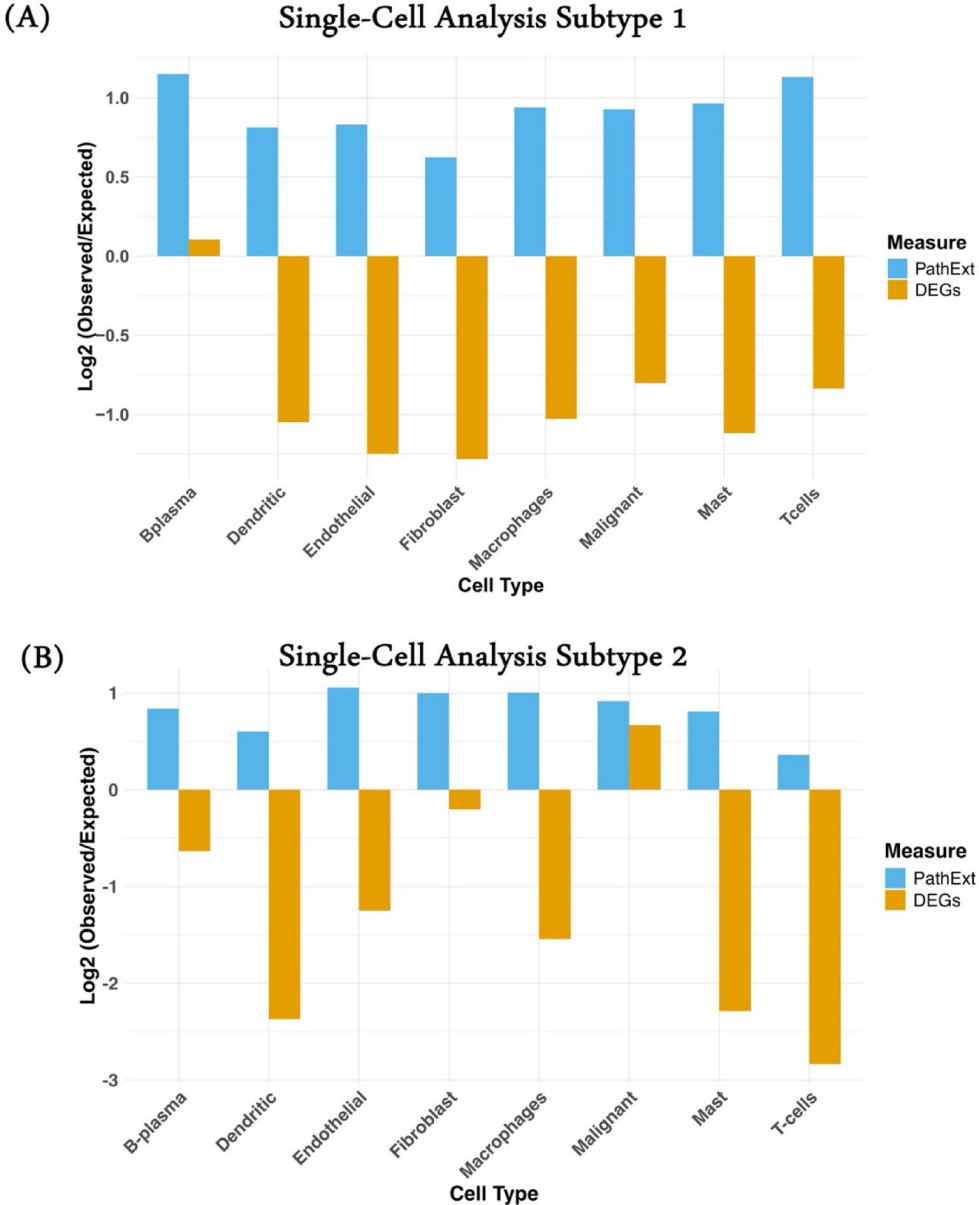
Single cell analysis. PathExt top100 central genes shows higher expression in cell types compared to upregulated top100 DEGs in (**A**) Subtype 1 and (**B**) Subtype 2.

Heterogeneity in HNSCC subtypes is attributable not only to variation in transcription in cancer cells but also to cellular composition in the tumor microenvironment. So, we looked at Choi et al.‘s HNSCC HPV-positive and negative single-cell transcriptome data to see how the top 100 genes (PathExt and DEGs) were expressed in the cells, obtained using bulk TCGA-HNSCC RNAseq data (see Methods). We first computed the mean expression and variance across cell types and performed one-way ANOVA for both DEG and PathExt gene sets. This showed that a majority of genes in both sets were significantly differentially expressed across cell types (*p* < 0.05), indicating cell type–specific expression variability in both (Supplementary Table S11). Therefore, we next performed z-scoring of each gene across cell types and then computed the logFC (observed/expressed) gene expression of genes in each cell type (Methods). We observed high expression of PathExt central genes above the expected level in both subtypes, highlighting its significant role in remodeling the TME, whereas in the case of DEGs, in subtype 1, only B-plasma (Fig. 5A) and in subtype 2, only malignant cells show the expression above the expected level (Fig. 5B). In subtype 1, PathExt central genes were frequently expressed in immune cells, i.e., B-plasma and T-cells, followed by malignant, macrophages, and mast cells (Fig. 5A), whereas in subtype 2, PathExt central genes were frequently expressed in endothelial cells, followed by fibroblasts, macrophages, and malignant cells (Fig. 5B).

### PathExt central genes classified responder and non-responder with high accuracy

**Fig. 6:**
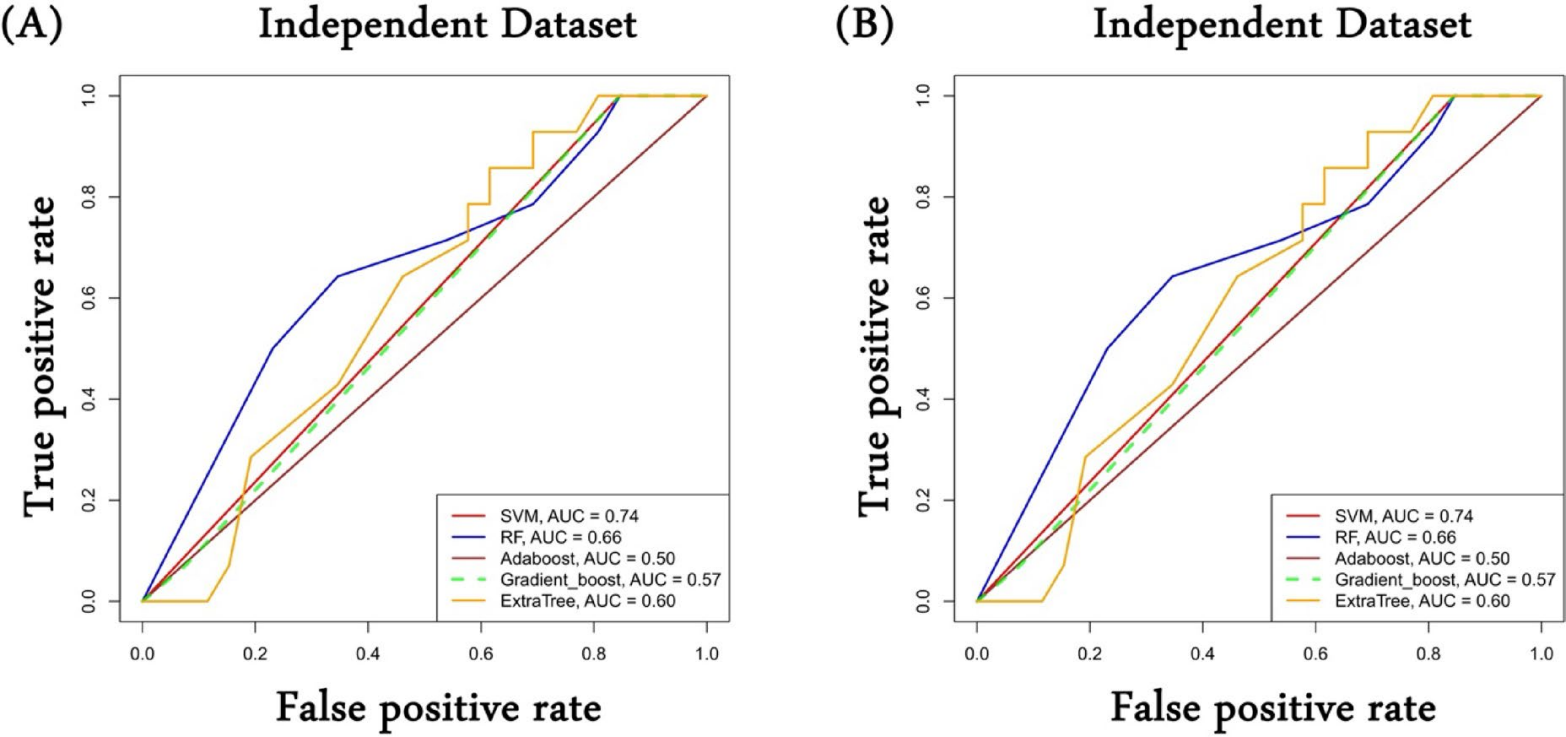
PathExt classifies responder and non-responder with high accuracy. Performance of machine learning models on independent dataset by (**A**) Subtype 2 PathExt central genes and (**B**) Subtype 2 DEGs. Performance measure is in the terms of AUROC.

We assessed the potential of PathExt central genes and DEGs in classifying the responders and non-responders were HNSCC patients were treated by cetuximab in a subtype-specific manner. In subtype 1, there were only 51 DEGs present for which the data was available in the validation dataset; hence we excluded evaluation of genes (PathExt and DEGs) for this subtype. For subtype 2, we had data for 99 PathExt central genes and 94 DEGs in the validation dataset. The model was trained for the same genes using the TCGA-HNSCC dataset (with HPV-negative as the positive class and HPV-positive as the negative class), and the performance was evaluated on the independent dataset. As shown in Fig. 6A, the PathExt gene-based SVM model has the highest AUROC of 0.74, followed by the RF-based model with an AUROC of 0.66; however, in the case of DEGs (Fig. 6B), a maximum AUROC of 0.52 was observed. The above observation shows that PathExt can capture the biology associated with patients responding to a treatment compared to DEGs.

To check the statistical significance of the model, we performed bootstrap resampling (*n* = 1000) on the held-out test set to compute 95% confidence intervals for AUROC. However, we observed relatively wide confidence intervals (CI), and non-significant bootstrap-based pairwise comparisons between classifiers. We also performed DeLong’s test for comparison of AUROC-based performance between ML models, however, due to smaller (40 samples) and imbalanced nature of dataset (14 positive and 26 negative), there was no significant observation. Therefore, to claim model performance, we used additional metrics such as AUPRC, and F1-score (Supplementary Table S12).

### Network analysis shared and unique therapeutic targets in HNSCC subtypes

The PPI network was created using STRING, and the interaction file was further imported into Cytoscape. We extracted the top 10 hub genes based on the ‘Degree’ node calculation method implemented in the ‘cytoHubba’ plugin. This analysis was performed for both subtypes, PathExt and top 100 PathExt central genes. We identified five shared targets: *JUN*,* SRC*,* CTNNB1*,* STAT3*, and *AKT1*. The other five targets were unique to each subtype. Next, we map these targets with the CMap database (https://clue.io/about), which provides the drug-target information. For each target, we proposed only three drugs. Please note, for some targets, only 1 or 2 drugs were present in the CMap database. Table 5 provides complete details of the targets, their interacting partners from Cytoscape, and potential drug lists associated with these targets.

**Table 5:** List of the subtype-specific targets and their potential drugs. This table comprises of top10 potential targets characterized in subtype manner. Maximum of 3 drugs are provided for each target after mapping with CMap data.

HNSCC Subtype 1			HNSCC Subtype 2		
Name	Score	Top 3 drugs	Name	Score	Top 3 drugs
TP53	46	APR-246, Carbendazim, Idasanutlin	JUN	38	Arsenic-trioxide, Irbesartan, Vinblastine
SRC	38	Bosutinib, Dasatinib, Ponatinib	EGFR	38	Afatinib, Axitinib, Brigatinib
AKT1	38	Arsenic-trioxide, Canertinib, Enzastaurin	SRC	37	Bosutinib, Dasatinib, Ponatinib
EP300	36	Curcumin, Epigallocatechin-gallate-(-), C646	CTNNB1	37	Urea, cardionogen-1, CCT-031374
JUN	35	Arsenic-trioxide, Irbesartan, Vinblastine	STAT3	36	Acitretin, Niclosamide, bardoxolone-methyl
ESR1	35	Allylestrenol, Clomifene, Desogestrel	FN1^*^	35	No data was present
STAT3	35	Acitretin, Niclosamide, bardoxolone-methyl	ACTB^#^	35	cytochalasin-b, ACTB-1003
HSP90AA1	33	Nedocromil, Rifabutin, Ganetespib	AKT1	34	Arsenic-trioxide, Canertinib, Enzastaurin
CTNNB1	31	Urea, cardionogen-1, CCT-031374	MYC	34	TWS-119
MDM2	30	Idasanutlin, JNJ-26,481,585, AMG-232	TGFB1	34	D-4476, F351

We also investigated the drug-gene interactions using DGIdb 5.0 database, which contains nearly 10,000 genes and 20,000 drugs involved in nearly 70,000 drug-gene interactions. In total, we obtained 1112 potential drug-gene interactions for subtype 1 and 636 potential drug-gene interactions for subtype 2. Further, we observed that TP53 showed highest maximum interaction with total of 452 in subtype 1, whereas in case of subtype 2, gene EGFR gene shows the maximum interaction of 192. Complete stats for both the subtypes are provided in the Supplementary tables S13 and S14.

We further investigated the previous literature to find the support for our findings. We observed role of proposed drugs against the target for several diseases. For example, Hangs et al. showed the drug APR-246 targets TP53 and GSTP1 for inducing selective cell death in HNSCC^52^. Similarly, activity of dasatinib against c-SRC has been shown in case of HNSCC^53,54^. Likewise, Jiang et al. exhibited the activity of niclosamide against STAT3 in HNSC^55^.

## Discussion

Tumor heterogeneity is one of the major issues in limiting the efficacy of successful cancer treatment outcomes. This has been observed in the HNSCC, where we have two distinct categories based on the HPV status: (i) HPV-Positive and (ii) HPV-Negative. It has been observed that HPV-Negative HNSCC patients are more difficult to treat compared to HPV-Positive HNSCC. PathExt, by identifying central mediators causing gene expression changes, has demonstrated its utility in refining the expression variance and is applicable in multiple translational contexts. This study aims to characterize the molecular distinctions between HNSCC HPV-Pos and HNSCC HPV-Neg samples.

In order to accomplish its objective, PathExt first identifies critical pathways—that is, those that show noticeably different activity in a particular transcriptome sample in comparison to controls—in a pre-defined knowledge-based gene network. Next, it identifies important mediators of the critical pathways. By doing this, PathExt depends more on network interactions and less on the highly variable^56^ differential expression of individual genes. It is possible to use PathExt on a single sample, so it can be used in clinical settings where large cohorts are less common than with differential expression approaches, which need adequate sampling. PathExt directly takes inter-sample heterogeneity into account by selecting important genes sample-wise (as opposed to a cohort for differential expression). In the current study, PathExt consistently identified genes that are not differentially expressed but mediate global transcriptomic changes of up to 34% and 50% in subtypes 1 and 2, respectively. Also, we observed 17 common genes among the top 100 PathExt central genes identified in subtypes 1 and 2, which reflects the shared processes, including the cell cycle, inflammation, immune response, etc., whereas in the case of DEGs, no common genes were seen.

We evaluated the performance of PathExt and DEGs in recapitulating the disease biology between the two subtypes. We started by using the top 100 genes to do a functional enrichment analysis. In subtype 1, PathExt genes were linked to immune-related and metabolic processes, such as “immune response-activating cell surface receptor,” “regulation of T-cell activation,” etc. HPV positive tumors are characterized by high infiltration of lymphocytes and higher antigen-specific responses, specifically involving cells like CD8^+^ T-cells, dendritic cells and B cells^57,58^. Enriched terms such as “Fc-gamma receptor signaling pathway involved in phagocytosis”, “lymphocyte differentiation”, “T cell costimulation” strengthens our findings. Also, the role of HPV-16 E7 oncoprotein in activation of EGFR–PI3K–AKT and NRF2, amplifying NFκB in driving tumor proliferation and survival is well known^59,60^, which was supported by the terms such as “phosphatidylinositol 3-kinase/protein kinase B signal transduction”, “canonical NF-kappaB signal transduction” in our finding.

In subtype 2, they were more linked to peptide-related processes, such as “peptidyl-serine phosphorylation” and “peptidyl-serine modification”. We investigated the relevance of these terms in our findings specifically enriched for HNSCC HPV negative patients and found sufficient independent studies supporting our above observation. For example, terms such as “peptidyl-serine phosphorylation”, “peptidyl-threonine phosphorylation”, “positive regulation of MAPK cascade”, etc. explains the biology where the activating mutations in RTKs, EGFR, PIK3CA, etc. has been observed, common in HNSCC HPV-negative patients. ERKs have been shown to directly phosphorylates serine/threonine residues on transcription factors such as c-Myc, CREB, etc. driving cell cycle progression (G1/S, G2/M) and survival^61–63^. Terms such as “receptor signaling pathway via STAT”, “interleukin-6 production”, and more provided the understanding of how immune evasion via STAT signaling takes place in HNSCC HPV negative patients. STAT3 has been found to be hyperactivated through cytokine such as IL-10, IL-6 stimulation or via EGFR mediated cross-talk^64^ .

In the case of DEGs, we observed enrichment of similar processes, such as “humoral immune response” and “defense response to bacterium,” in both subtypes 1 and 2. This observation highlights the ability of the PathExt in identifying distinct genes enriched for distinct processes. Next, we looked at how much PathExt central genes and DEGs from both subtypes overlapped with the gene signatures linked to driving cancer in HNSCC. We saw a lot of overlap between PathExt genes and the driver genes, with a high odds ratio and FDR ≤ 0.05, but not much overlap between DEGs and the driver genes. We observed a similar pattern with the TSGs and the FDA-approved drug target list. We further look for the cancer hallmark enrichment and observed significant enrichment for PathExt-associated PathExt central genes compared to DEGs, which show enrichment for very few hallmarks (a maximum of 5). Likewise, we compared the enrichment of the gene sets (PathExt and DEGs) in the 395 mutated PPI systems retrieved from the NeST database and observed high significant enrichment for PathExt genes in both subtypes compared to DEGs, which show enrichment only in subtype 2, and that too very few. Comparative performance of PathExt with another network-based method, MOMA showed the superiority of the PathExt based approach where the overlap analysis shows high Odds ratio and significant p-values as per Fisher’s exact test.

PathExt was able to capture upstream regulators such as TFs and kinases in the top100 genesets compared to DEGs significantly. These findings were also supported by the GO terms related to transcription and kinase activity for both the subtypes, however in case of DEGs no such terms were observed.

The analysis of a single cell showed that PathExt genes were expressed significantly higher than expected in all cell types. This was in contrast to DEGs, where gene expression was lower than expected in most cell types. Machine learning analyses shows that ML models developed using PathExt based key expression predicts responder and non-responder with higher AUROC compared to DEGs. Though as per bootstrap sampling and DeLong’s test the results were not significant due to lower size and class imbalanced nature of the dataset, incorporating more such studies with balanced class size will improve the reliability and performance of the model in future studies.

Lastly, we created a PPI interaction network using STRING and characterized the top 10 hub genes using the ‘cytoHubba’ plugin in Cytoscape, which probably have potential therapeutic value. These 10 targets were mapped to the CMap database, and the top three drugs associated with them were proposed.

Overall, we observed PathExt as a complementary approach to DEGs, characterizing common and HNSCC subtype-specific PathExt central genes associated with distinct molecular subtypes. Here, we implemented PathExt in a specific context, namely, HNSCC HPV subtypes; however, the method holds potential application for generally identifying PathExt central genes associated with any clinical features. For instance, using a deconvolution approach, we can identify the various cell types, such as macrophages, in each HNSCC sample. We can implement our PathExt approach to characterize PathExt central genes between samples with high and low macrophage gene expression.

## Limitations of the study

The current study has few limitations, summarized as follows. Firstly, the current study relies only on one single bulk RNA-sequencing and single cell dataset. We extensively searched publicly available datasets across GEO, ArrayExpress, and other databases, however, we didn’t find datasets with transcriptomic data along with annotation of HPV status in HNSC patients. Secondly, current ML models for classifying responder and non-responder to given treatment was developed and validated using limited and highly imbalanced dataset. This was also shown in our statistical analysis when we implemented bootstrapping and DeLong test. Hence, more such datasets are required to developed robust and efficient models. Thirdly, the current study is purely computational and lacks experimental validation. Future studies require to validate these findings and function of key PathExt-identified genes using qPCR, siRNA knockdowns, and functional assays in HPV + and HPV– HNSCC cell lines.

## Supplementary Information

Below is the link to the electronic supplementary material.

Supplementary Material 1

Supplementary Material 2

## Data Availability

This paper analyzes existing, publicly available data which can be downloaded from the TCGA and GEO. All the codes, datasets used to develop the machine learning models for each class is provided at out GitHub repository accessible at https://github.com/agrawalpiyush-srm/HNSCC-HPVProject. Further information can be provided upon reasonable request to the corresponding author.
