# Supplementary material for: Network-based approach identifies key genes associated with tumor heterogeneity in HPV positive and negative head and neck cancer patients
Source: Sci Rep. 2025 Aug 7;15:28864. doi: 10.1038/s41598-025-13604-0 (PMC12332169; doi:10.1038/s41598-025-13604-0)
Supplement: Supplementary file 1 — Supplementary Material 1 [file 41598_2025_13604_MOESM1_ESM.docx]

**Network-based approach identifies key genes associated with tumor heterogeneity in HPV positive and negative head and neck cancer patients**

Sumeet Patiyal^1^ and Piyush Agrawal^2^*

1. Cancer Data Science Laboratory, National Cancer Institute, NIH, Bethesda, MD 20814, USA
2. Division of Medical Research, SRM Medical College Hospital and Research Centre, SRM Institute of Science and Technology, Kattankulathur, Chennai.

* Corresponding author

**Corresponding Author Details**

Piyush Agrawal. Ph.D.

**Address:** Division of Medical Research, SRM Medical College Hospital and Research Centre, SRMIST, Kattankulathur, Chennai

**Email:** [piyusha@srmist.edu.in](mailto:piyusha@srmist.edu.in), [apiyush74@gmail.com](mailto:apiyush74@gmail.com)

**ORCID:** 0000-0003-2075-1111


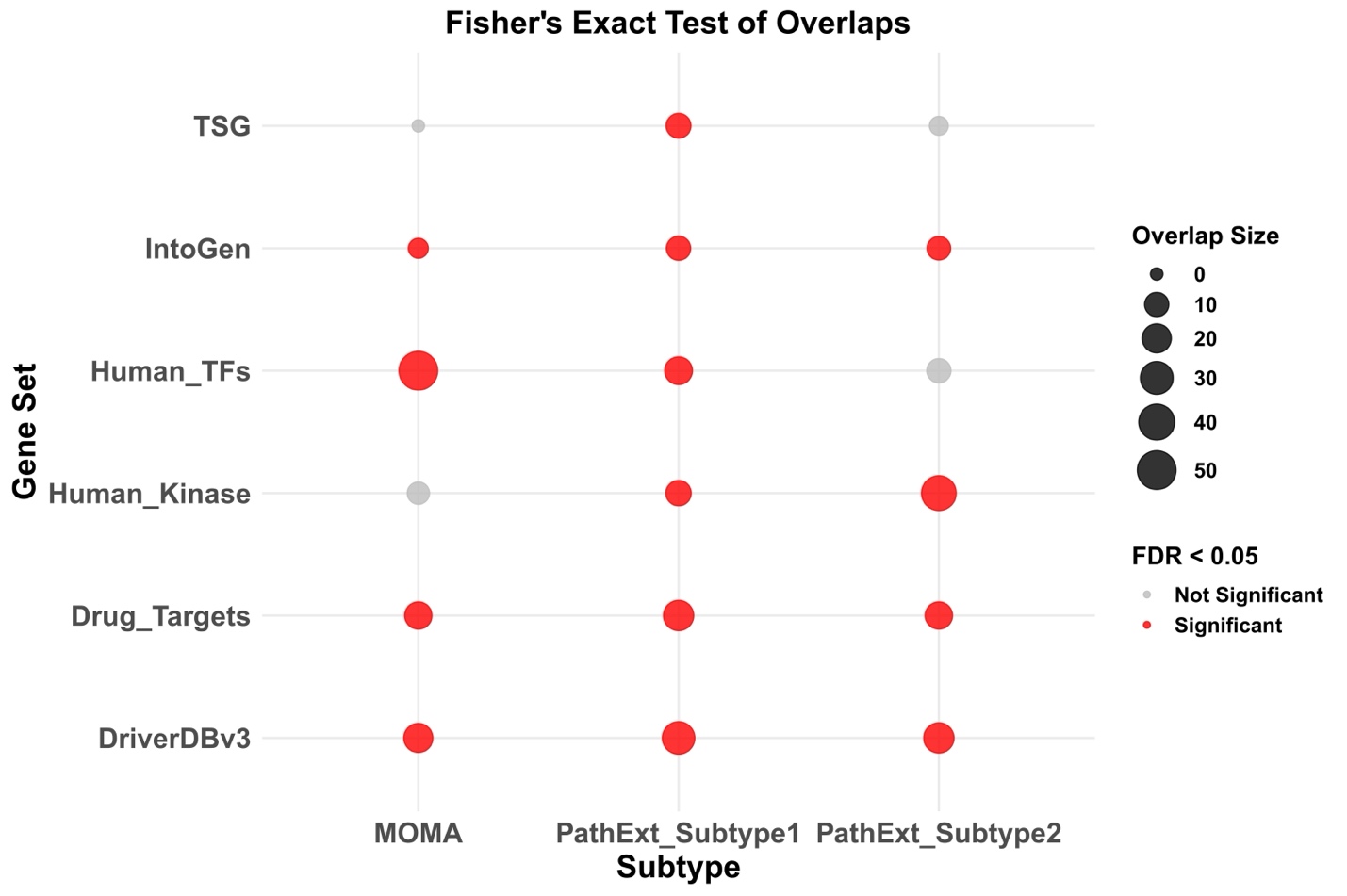


**Supplementary Figure S1. Comparative analysis of PathExt with MOMA.** Overlap analysis of 140 head and neck specific master regulators from MOMA with top100 genes form PathExt subtypes were performed. Fisher’s exact test was implemented, and odds ratio (OR) was computed. Dot represents the size of OR, red color signifies significant overlap whereas grey color signifies non-significant overlap.


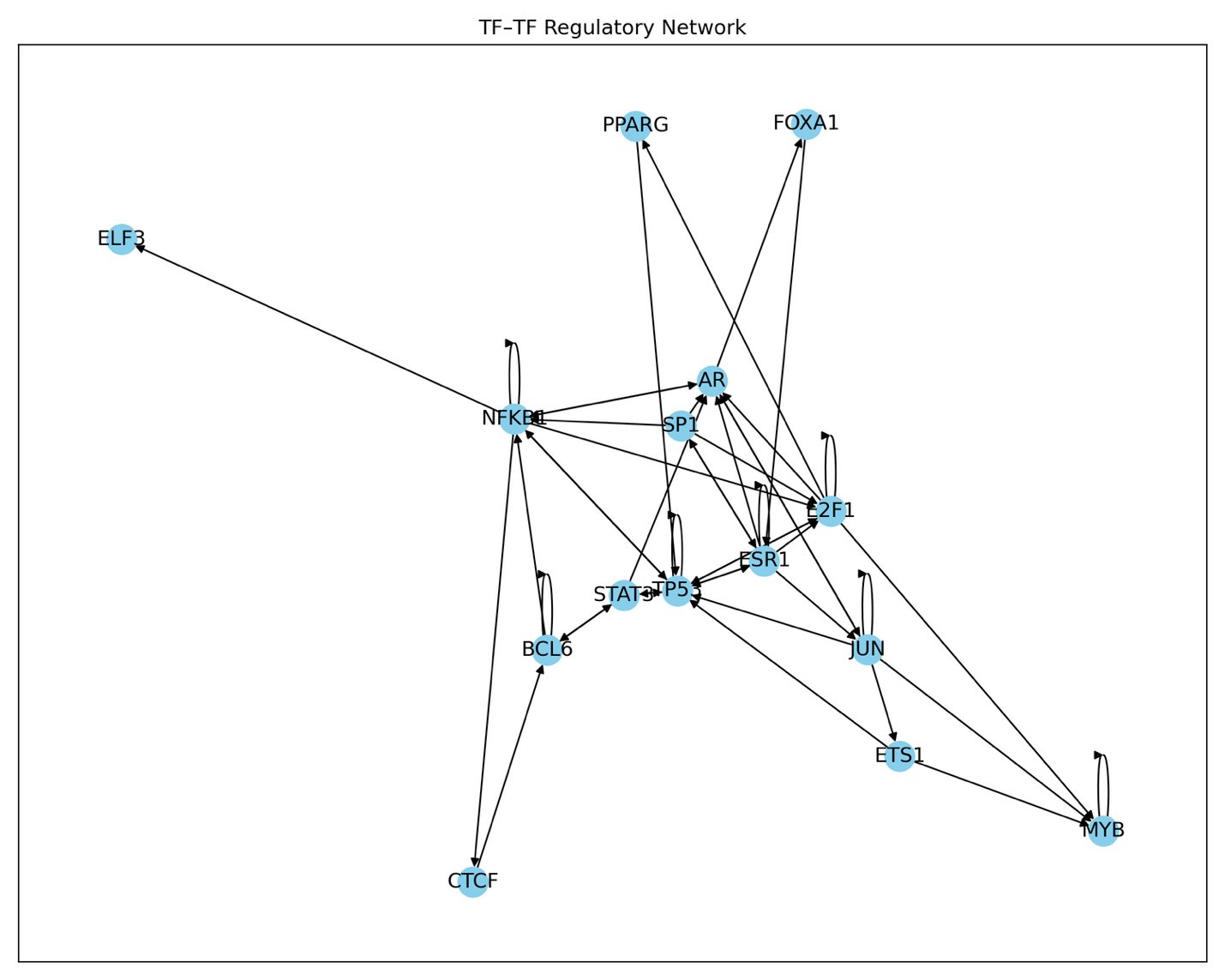


**Supplementary Figure S2.** TF-TF regulator network was created using Trrust tool. The network was created using TFs present in PathExt top100 genes in subtype 1.


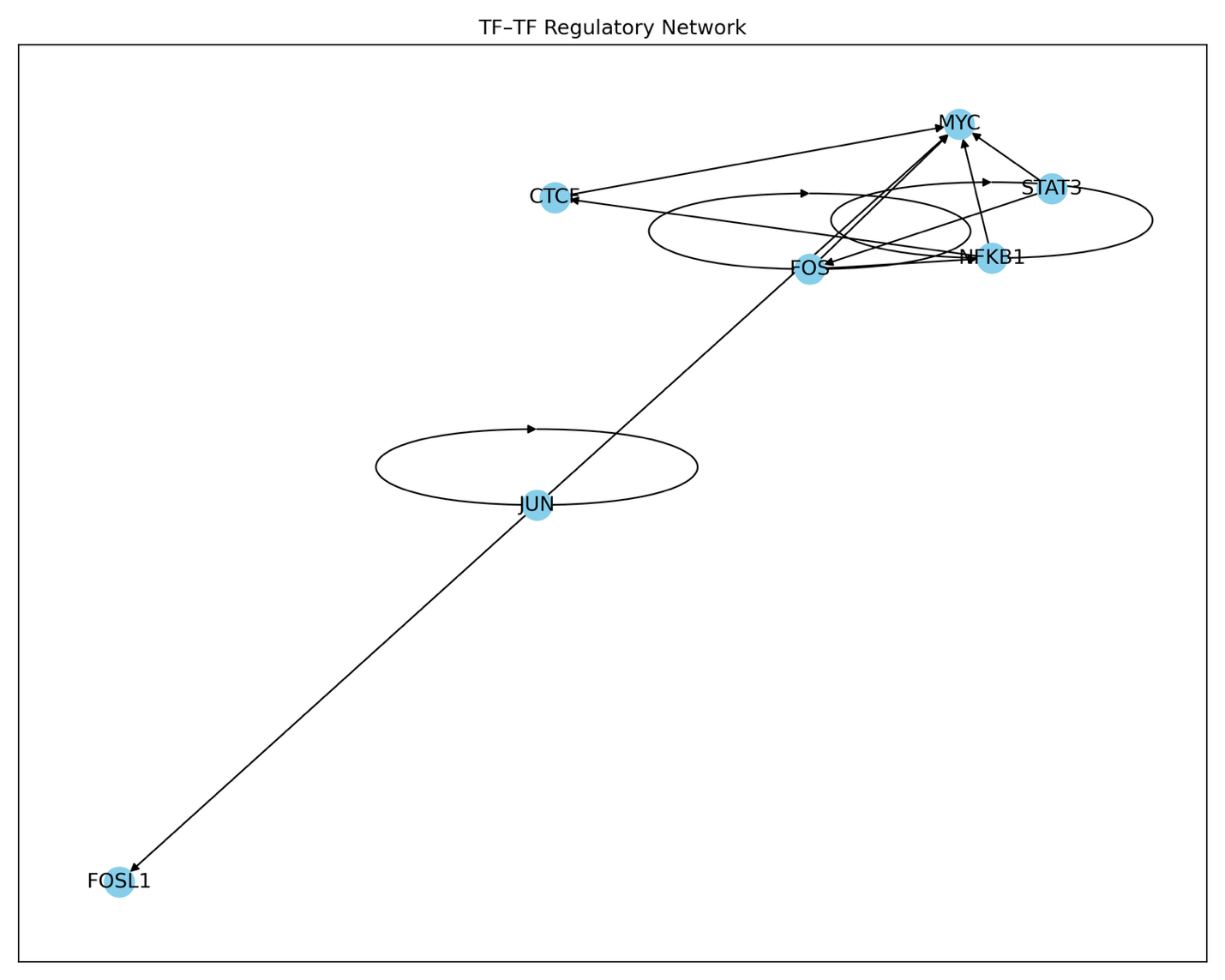


**Supplementary Figure S3.** TF-TF regulator network was created using Trrust tool. The network was created using TFs present in PathExt top100 genes in subtype 2.
